# Supplementary material for: The genome sequence of the Royal Mantle, Catarhoe cuculata (Hufnagel, 1767) (Lepidoptera: Geometridae)
Source: Wellcome Open Res. 2026 Jun 25;11:383. [Version 1] doi: 10.12688/wellcomeopenres.26829.1 (PMC13555075; doi:10.12688/wellcomeopenres.26829.1)
Supplement: Peer review history

# Reviewer report: Reviewer response for version 1

**DOI:** 10.21956/wellcomeopenres.29543.r159523

**Published:** 2026-07-14

**Authors:** Ranjana Bhaskar

**Affiliations:** 1 Southern Regional Centre, Zoological Survey of India, Chennai, Tamil Nadu, India

The authors have presented a comprehensive whole-genome assembly of Catarhoe cuculata a Lepidopteran species. Two haplotypes were identified, and a high-quality de novo genome assembly was generated using PacBio long-read sequencing. The assembly comprises 31 chromosome-scale scaffolds, including the Z sex chromosome. Overall, the authors have provided sufficient details regarding the genome assembly, and the methodology and assembly statistics are clearly described.

Are sufficient details of methods and materials provided to allow replication by others?

Yes

Is the rationale for creating the dataset(s) clearly described?

Yes

Are the datasets clearly presented in a useable and accessible format?

Yes

Are the protocols appropriate and is the work technically sound?

Yes

Reviewer Expertise:

Molecular texonomy, Conservation genetics, population studies

I confirm that I have read this submission and believe that I have an appropriate level of expertise to confirm that it is of an acceptable scientific standard.

# Reviewer report: Reviewer response for version 1

**DOI:** 10.21956/wellcomeopenres.29543.r159520

**Published:** 2026-07-09

**Authors:** Mohadeseh Tahami

**Affiliations:** 1 University of Jyväskylä, Jyväskylä, Finland

The study presents the whole genome assembly of *Catarhoe cuculata *butterfly, local to the Great Britain and Ireland. The species was identified both morphologically and using DNA barcoding against the BOLD database. De novo genome assembly was performed using Long-read PacBio technology and Hi-C was used for verifying chromosome scaffolds. The sequencing data provided x138 coverage and the BUSCO completeness for both haplotypes were 98.4%.

 The material and methods and the statistics are in details however, authors could have still explained better the rationale of the methodology used.

 1. I missed to understand whether this is the first genome assembly of *C.* *cuculata *species or are there other assemblies in the databank either partial or using older techniques?

 2. The text quickly jumps to the sequencing methods without mentioning what technology is used for the genome assembly and why those are used. This could be added to the last paragraph of the background or in the beginning of the method section.

 3. The manuscript could benefit from clarifying why the authors chose haplotype one for chromosome level assembly, have they run comparison assembly between the two haplotypes? Are the two haplotypes covering same regions of the genome, in other words, what percentage of the two haplotypes match?

 All in all, the work is presented well, using a combination of long-read and Hi-C technology yielded a robust result.

Are sufficient details of methods and materials provided to allow replication by others?

Yes

Is the rationale for creating the dataset(s) clearly described?

Partly

Are the datasets clearly presented in a useable and accessible format?

Yes

Are the protocols appropriate and is the work technically sound?

Yes

Reviewer Expertise:

evolutionary genomics, NGS analysis

I confirm that I have read this submission and believe that I have an appropriate level of expertise to confirm that it is of an acceptable scientific standard.
